# Isolated subacute combined degeneration in late-onset cobalamin C deficiency in children: Two case reports and literature review

**DOI:** 10.1097/MD.0000000000017334

**Published:** 2019-09-27

**Authors:** Junling Cui, Yuanyuan Wang, Huifeng Zhang, Xiaopu Cui, Lihui Wang, Huacheng Zheng

**Affiliations:** a Department of Neurosurgery, The Second Hospital of Hebei Medical University; b Second Department of Neurology, The Children's Hospital of Hebei Province; c Department of Pediatrics, The Second Hospital of Hebei Medical University, Hebei Medical University, Shijiazhuang, Hebei, China.

**Keywords:** CblC disorder, child, homocysteinemia, subacute combined degeneration, vitamin B12

## Abstract

**Rationale:**

Subacute combined degeneration (SCD) is a disease caused by decreased vitamin B12 intake or metabolic disorders. It is more common in the elderly and rarely seen in children. Here, we report 2 pediatric cases of SCD in late-onset cobalamin C (CblC) deficiency.

**Patient concerns:**

The patients complained of unsteady gait. Their physical examination showed sensory ataxia. Magnetic resonance imaging showed classic manifestations of SCD. The serum vitamin B12 level was normal, but urine methylmalonic acid and serum homocysteine levels were high.

**Diagnosis:**

The pathogenic gene was confirmed as *MMACHC*. The 2 patients each had 2 pathogenic mutations C.482 G>A and C.271dupA and C.365A>T and C.609G>A in this gene. They were diagnosed with combined methylmalonic acidemia and homocysteinemia-CblC subtype.

**Interventions:**

The patients were treated with methylcobalamin 500 μg intravenous injection daily after being admitted. After the diagnosis, levocarnitine, betaine, and vitamin B12 were added to the treatment.

**Outcomes:**

Twelve days after treatment, the boy could walk normally, and his tendon reflex and sense of position returned to normal. The abnormal gait seemed to have become permanent in the girl and she walked with her legs raised higher than normal.

**Lessons:**

To the best of our knowledge, this is the first report of 2 cases of isolated SCD in children with late-onset CblC disorder. Doctors should consider that SCD could be an isolated symptom of CblC disorder. The earlier the treatment, the lower the likelihood of sequelae.

## 1 Introduction

Subacute combined degeneration (SCD) is caused by the deficiency of vitamin B12 intake, absorption, transit, metabolism, or a combination of these. Nitrous oxide (N_2_O) and a decreased copper level can also induce SCD. Vitamin B12 deficiency leads to central and peripheral nervous system demyelination disease, especially in the posterior columns of the spinal cord and peripheral nerve. Testing for serum vitamin B12 and methylmalonic acid levels and the characteristic magnetic resonance imaging (MRI) findings can assist in the diagnosis of SCD.^[1,2]^ Vitamin B12 is produced by bacteria in the gut, and diet and intestinal diseases causing its deficiency are considered as exogenous factors. SCD is more common in the elderly, but rare in children. The commonest underlying reason is the inability to absorb dietary cobalamin in the elderly, but in children, the reason is different.

This paper mainly discusses the diagnostic process for 2 pediatric SCD cases with late-onset cobalamin C (CblC) disorder.

## 2 Consent

This was a retrospective study, without any intervention treatment or examination; hence, the ethics committee approval was not obtained. Informed written consent was obtained from each patient's for publication of their case report and accompanying images.

## 3 Case report

### 3.1 Case 1

A 13-year-old girl presented to a local hospital in December 2015, with a 2-week history of unsteady walking and unwillingness to communicate with others. Her brain MRI showed a slight widening of the sulci; electroencephalogram (EEG) showed slow background activity. During the 2 weeks without treatment, her condition remained unchanged. She was transferred to our hospital 3 days after the initial presentation to the local hospital. Her family history was unremarkable, and she was the third child of her family. She did not like studying, but liked sports. Both her height and weight were normal. Physical examination revealed hypoesthesia, inability to perform fine movements with the hands, unwillingness to interact with other human beings, and a normal sleep-wake cycle.

Laboratory findings were as follows: normal hemoglobin level; the levels of vitamin B12 measured twice with a 1-week interval were 610.10 pg/mL and 734.10 pg/mL; the folic acid level was 8.05 ng/mL. The bilateral lower limb sensory evoked potential showed abnormalities (central segment). Cerebrospinal fluid routine examination was normal. MRI of the neck showed symmetrical long T2 signals in the posterior portion of the spinal cord in the 2 to 6 vertebral bodies. Urinary methylmalonic acid and serum propionyl-carnitine C3/C2 levels were elevated. The serum homocysteine level was 133.4 μmol/L (normal adult 0–15 μmol/L).

The patient and her family agreed to the *MMACHC* gene test at JinYu Medical Laboratory. The test confirmed 2 known pathogenic heterozygous mutations, C.482 G>A and C.271dupA. The C.271dupA mutation was passed on from the father, and C.482 G>A from the mother. Her 2 siblings have only 1 pathogenic heterozygous mutation for the disease.

Since admission, 500 μg methycobal was injected every day. After the diagnosis, L-carnitine 1 g twice a day, intramuscular injection of vitamin B12 twice a week, and betaine orally were added to the treatment. Two months later, she could walk with her legs slightly raised and her communication skills returned to normal, but 2 years later, she still exhibited abnormal gait.

### 3.2 Case 2

A 6-year-old boy was admitted to the Neurology Department in August 2017 because of unsteady walking for 6 days. He had shown no obvious abnormality before that; during those 6 days, he lifted his leg high while walking, showed wide-based gait, and fell easily. He could walk alone for a few steps. His previous and personal history was as follows: he was the second child in his family. He had normal birth weight, head circumference, height, and developmental history. He was a mediocre pupil in grade 1 and liked sports. He was not picky about food and had a normal physique for his age. Two years ago, his older sister was admitted to a local hospital because of weakness in both lower extremities; her condition normalized 2 weeks later, after being treated with vitamin B, immunoglobulin, and glucocorticoid administration. The boy's physical examination revealed the following: normal body development, a clear mind, and good reaction. Cardiopulmonary and abdominal examinations were normal. No abnormalities were observed on cranial nerve examination. The muscle strength of his limbs was normal, but the muscle tone of his lower limbs was low. Bilateral knee tendon reflex was decreased. His sensations of pain and heat were normal. The joint position sense of the lower limbs was impaired; he did not cooperate for the vibration sense investigation. Bilateral Babinski reflex was normal. Upper limb rotation and finger-to-nose tests were normal, but the heel-to-shin and Romberg tests were abnormal.

Laboratory findings were as follows: hemoglobin 9.9 g/dL and mean corpusular volume 84.8 fL. He showed positivity for anti-SS-ARo 54 kd and anti-SS-ARo 60 kd, which became normal 1 week later. The antithyroid peroxidase antibody level was 488.6 IU/mL. The lactic acid level was 6.74 mmol/L. Blood ammonia and copper levels were normal. The levels of vitamin B12 (882.5 pg/mL) and folic acid were normal. Chest radiography, electrocardiograph, and EEG were normal. Cerebrospinal fluid routine examination was normal, except that the lactic acid level was 3.43 mmol/L.

**Figure 1:**
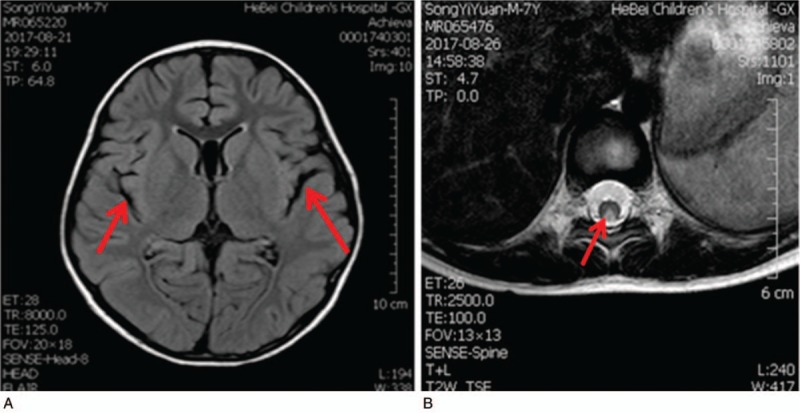
MRI findings of the patient. (A) The enlargement of the cerebral groove in the skull MRI. (B) The axial position of the thoracic segment shows the hyperintense T2W1 signals in the posterior columns, presenting an inverted “V” sign. MRI = magnetic resonance imaging. T2W1 = T2 weighted image.

His brain MRI showed a right choroid cyst, the cavity of septum pellucidum, and slightly wider sulci (Fig. 1A). MRI of the spinal cord revealed an abnormally long T2 weighted image (T2W1) signal in the posterior columns from T8 to T11 (Fig. 1B). Urinary methylmalonic acid was 216.7 (normal 0–4); propionyl-carnitine C3 was 10.15 μmol/L (normal 0.3–5 μmol /L); C3/C2 was 1.05/1 (normal 0.02–0.25); methionine was 20.80 μmol/L (normal 8–50 μmol/L); and serum homocysteine was 44.552 μmol/L (normal adult 0–15 μmol/L).

**Figure 2:**
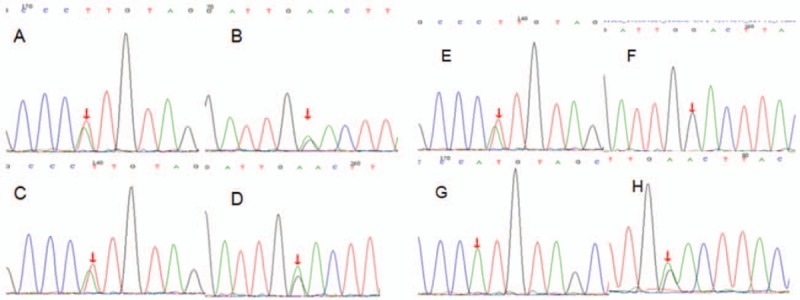
Mutations detected in the *MMACHC* gene test in second patient. (A) Patient c.365A>T, (B) patient c.609G>A, (C) sister c.365A>T, (D) sister c.609G>A, (E) father c.365A>T, (F) no mutation in father, (G) no mutation in mother, (H) mother c.609G>A.

The patient and his mother agreed to the *MMACHC* gene test at Beijing MyGenostics Medical Laboratory. The test confirmed the presence of 2 known pathogenic heterozygous mutations, C.365 A>T and C.609G>A. Later, blood samples of the older sister and father were collected, which revealed that the older sister had the same mutations as her brother. The C.365>A mutation was passed on from the father and C.609G>A from the mother (Fig. 2).

Demyelinating myelitis could not be excluded at the beginning and, hence, immunoglobulin treatment was prescribed. Additionally, 500 μg methycobal was injected every day. After diagnosis, L-carnitine 1 g twice a day was added to the treatment. Twelve days after treatment, the patient could walk normally, and his tendon reflex and sense of position returned to normal. The T2W1 signal on MRI was slightly lower. Vitamin B12 administration was effective. After discharge from the hospital, vitamin B12 and betaine treatments were added. A normal diet was maintained. After 6 months of treatment, the boy had no complaints.

Both children had normal serum levels of vitamin B12. However, the levels of methylmalonic acid, C3, and homocysteine suggested endogenous vitamin B12 deficiency. The MRI findings resembled those of SCD with an inverted “V” sign in the posterior columns, consistent with the diagnosis of SCD. After genetic examination, the patients were diagnosed as having late-onset CblC disorder of methylmalonic acidemia (MMA).

## 4 Discussion

We reported 2 pediatric cases of SCD in late-onset CblC deficiency. CblC deficiency is an endogenous vitamin B12 deficiency disease. Vitamin B12 was finally isolated in the mid-20th century; this greatly improved the associated neurological manifestations.^[2]^ It is mainly consumed from diet and then converted by gut microorganisms into 2 active forms, methylcobalamin (MeCbl) and adenosylcobalamin (AdoCbl). A disorder of AdoCbl enhances accumulation of methylmalonic acid and related enzymes (including methyl citrate, malonic acid, and propionyl CoA), which damage the mitochondrial metabolism of organs, especially the central nervous system, which then leads to poor removal of these accumulated components. A lack of MeCbl increases homocysteine levels, which in turn, decreases methionine levels and results in megaloblastic anemia.^[3]^ The pathogenesis of SCD is still unclear. Defective transmethylation of myelin is known as the direct cause of SCD, also referred to as SCD due to a dysfunction of the methyl-transfer pathway.^[4]^ As most cases of SCD occur in the elderly who do not undergo the tests for inherited metabolic disorders, the levels of methionine in such cases have remained unknown. Therefore, the pathogenesis cannot be inferred from clinical laboratory results.

SCD is rare in children and adolescents. Moreover, SCD has never been reported in China among children. We searched for the keywords “Subacute combined degeneration” and “child” in all fields in PubMed; since the establishment of the library to 2019–3, there were only 33 articles; excluding those without detailed records, with inconsistent content, and review of literature, 11 articles with 20 cases were obtained (including our 2 cases, Table 1).^[3–13]^ Of the 20 cases, 2 patients were vegans, 2 cases were caused by chemotherapeutics, and 1 case was of early-onset CblC disorder, which was identified by routine newborn urine screening. Further, 2 cases were due to vitamin B12 metabolism diseases, 5,10-methylenetetrahydrofolate reductase deficiency, and Grasbeck–Imerslund syndrome. In 3 cases, the etiologies were not mentioned, and the other 8 cases were all affected by exposure to N_2_O; however, since it is known that CblC disorder can also be affected by exposure to N_2_O, in the 11 cases, CblC disorder was not ruled out, especially in the 3 cases with nuclear etiologies.

**Table 1:**
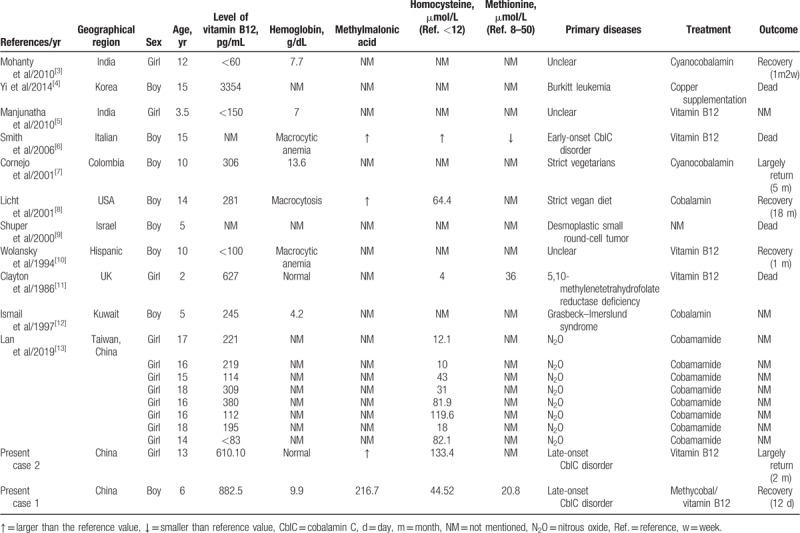
Cases of children with subacute combined degeneration in the literature.

Patients with early-onset CblC disorder are more likely to present with symptoms within 1 year of age, and most of them involve multiple organs, which is relatively easy to identify compared to late-onset CblC disorder. Smith et al reported a case of early-onset; the patient's clinical features included developmental delay and megaloblastic anemia since birth. By 9 years of age, he became unsteady and nonambulatory, and by 15 years of age, he had developed distal extremity atrophy and died after multiple vomiting episodes. In the autopsy, the thoracic segments showed demyelinating signs in multiple typical sites and vacuolation, and the axon was relatively intact, with macrophage infiltration and increased reactive astrocytes, mostly focused on the lateral column and posterior funiculus of the middle thoracic cord. All these features conform to the pathological manifestations of SCD.^[6]^ Thus, CblC disorder can present as SCD. In summary, the examination of vitamin B12 in these 20 patients was not complete, and more attention was paid to the identification of exogenous causes, and less attention was paid to endogenous vitamin B12 deficiency.

There are 2 reported cases of isolated SCD as the first clinical feature in adult late-onset CblC disorder. In 2008, a 42-year-old man with normal vitamin B12 level was diagnosed with SCD secondary to CblC disorder.^[14]^ In 2014, a 26-year-old man with SCD was diagnosed, 4 years after initial symptom onset, due to additional complaints about cognitive impairment and thrombosis; his vitamin B12 level was also normal.^[15]^ In these 2 cases, SCD was the first symptom, and later mental abnormality symptoms and thrombosis occurred, which delayed the diagnostic process and almost returned to normal after the conventional CblC disorder treatment. Considering these 2 cases and our cases, the serum vitamin B12 levels were normal in all 4 cases of late-onset CblC disorder. Although many reports regarding SCD indicate that the vitamin B12 level is normal in a small number of enteric-SCD patients,^[2]^ these reports do not completely exclude the possibility of secondary SCD; hence, genetic testing is necessary for patients when encountering SCD with normal vitamin B12 levels.

The incidence of MMA in the United States is 1 in 100,000 persons, while in Taiwan, 14 patients have MMA among 1,321,123 newborns.^[16]^ The CblC type is the most common type in China.^[17]^ C.271dupA was the most frequently mutated allele in late-onset CblC disorder patients. In our report, in case 2, there were 2 loci of heterozygous mutations: C.609G>A (p.W203X), a nonsense mutation, which was derived from the mother, and C.365A>T (p.H122L), a missense mutation, which changed the amino acid from histidine to leucine, derived from the father. C.609G>A is the most common mutation in China, and currently, the mutation has only been shown to occur in Asians. C.365A>T was first reported in China, and no other countries have reported it subsequently.^[18]^ Late-onset CblC disorder is difficult to diagnose because of its clinical variability. Many patients can only be diagnosed after years of irreversible damage. Since increased methylmalonic acid and homocysteine levels occur both in SCD and CblC disorder, genetic diagnosis can help diagnose MMA.

This report is limited because the number of cases included was small, and some experimental data of the first patient were not documented; thus, more cases need to be collected and analyzed on this topic.

In summary, late-onset CblC disorder affects many organs and its clinical manifestations are varied. There is a huge variability in its age at occurrence and it can even occur as late as in the 40s.^[14,19]^ In the second patient in this report, all abnormality associated with this disease was resolved because of the timely diagnosis and treatment. On the contrary, the first patient visited the doctor 1 week later than the second patient and has sustained an abnormal walking posture. For SCD or late-onset CblC disorder, earlier recognition and treatment can result in complete reversal of the damage.

## Acknowledgments

The authors thank the patients and their families for their cooperation.

## Author contributions

**Conceptualization:** Junling Cui.

**Formal analysis:** Huifeng Zhang.

**Funding acquisition:** Yuanyuan Wang.

**Investigation:** Huifeng Zhang, Lihui Wang.

**Project administration:** Junling Cui, Xiaopu Cui.

**Resources:** Lihui Wang.

**Supervision:** Huacheng Zheng.

**Visualization:** Huifeng Zhang, Xiaopu Cui, Lihui Wang.

**Writing – original draft:** Yuanyuan Wang.

**Writing – review and editing:** Junling Cui, Huacheng Zheng.

Yuanyuan Wang orcid: 0000-0002-2114-0980.
